# Invasive *Nocardia* Infections across Distinct Geographic Regions, United States

**DOI:** 10.3201/eid2912.230673

**Published:** 2023-12

**Authors:** Simran Gupta, Leah M. Grant, Harry R. Powers, Kathryn E. Kimes, Ahmed Hamdi, Richard J. Butterfield, Juan Gea-Banacloche, Prakhar Vijayvargiya, D. Jane Hata, Diana M. Meza Villegas, Adrian C. Dumitrascu, Dana M. Harris, Razvan M. Chirila, Nan Zhang, Raymund R. Razonable, Shimon Kusne, Salvador Alvarez, Holenarasipur R. Vikram

**Affiliations:** Mayo Clinic Arizona, Phoenix, Arizona, USA (S. Gupta, L.M. Grant, K.E. Kimes, R.J. Butterfield, J. Gea-Banacloche, N. Zhang, S. Kusne, H.R. Vikram);; Mayo Clinic Jacksonville, Jacksonville, Florida, USA (H.R. Powers, D.J. Hata, D.M. Meza Villegas, A.C. Dumitrascu, D.M. Harris, R.M. Chirila, S. Alvarez);; Mayo Clinic Rochester, Rochester, Minnesota, USA (A. Hamdi, P. Vijayvargiya, R.R. Razonable)

**Keywords:** Nocardia, nocardiosis, bacteria, transplant, transplantation-associated infections, environmental pathogens, disseminated, invasive infections, United States

## Abstract

We reviewed invasive *Nocardia* infections in 3 noncontiguous geographic areas in the United States during 2011–2018. Among 268 patients with invasive nocardiosis, 48.2% were from Minnesota, 32.4% from Arizona, and 19.4% from Florida. Predominant species were *N. nova* complex in Minnesota (33.4%), *N. cyriacigeorgica* in Arizona (41.4%), and *N. brasiliensis* in Florida (17.3%). Transplant recipients accounted for 82/268 (30.6%) patients overall: 14 (10.9%) in Minnesota, 35 (40.2%) in Arizona, and 33 (63.5%) in Florida. Manifestations included isolated pulmonary nocardiosis among 73.2% of transplant and 84.4% of non–transplant patients and central nervous system involvement among 12.2% of transplant and 3.2% of non–transplant patients. *N. farcinica* (20.7%) and *N. cyriacigeorgica* (19.5%) were the most common isolates among transplant recipients and *N. cyriacigeorgica* (38.0%), *N. nova complex* (23.7%), and *N. farcinica* (16.1%) among non–transplant patients. Overall antimicrobial susceptibilities were similar across the 3 study sites.

*Nocardia* is a genus of aerobic, filamentous, beaded, gram-positive bacteria ubiquitous in the environment, especially in soil, decomposing organic matter, and water. *Nocardia* spp. are opportunistic pathogens in immunocompromised hosts and immunocompetent persons with chronic lung disease (*1*). The Centers for Disease Control and Prevention has estimated that the United States sees 500–1,000 new cases of *Nocardia* infection annually (*2*,*3*). Pulmonary infections acquired by inhaling aerosolized organisms account for most *Nocardia*-associated illness (*4*). Direct inoculation, which causes cutaneous infections, constitutes the second most common route of exposure (*3*,*5*). Patients with defects in cell-mediated immunity are at highest risk for infection (*6*,*7*). Other risk factors include systemic corticosteroid use, solid organ or hematopoietic stem cell transplantation, HIV infection, diabetes mellitus, and underlying malignancy being treated with chemotherapy (*8*). The brain, skin, and soft tissues, and to a lesser degree bones and joints or other organs, comprise the most common sites of extrapulmonary dissemination (*6*,*9*).

Geographic distribution of various *Nocardia* spp. and their effect on human disease has not been described. Although *N. cyriacigeorgica* (formerly *N. asteroides* drug pattern type VI) (*10*,*11*) and *N. farcinica* are distributed evenly throughout the United States, distribution of other *Nocardia* spp. varies by geographic location. *N. brasiliensis* is associated with tropical and subtropical environments and has a higher prevalence in the southwestern and southeastern regions of the United States (*4*,*12*). Because Mayo Clinic, headquartered in Rochester, Minnesota, USA, operates tertiary locations in the midwestern, southeastern, and southwestern United States, we had a unique opportunity to study *Nocardia* infections in those 3 distinct noncontiguous geographic areas. 

## Methods

We performed a multicenter retrospective cohort study of patients evaluated at Mayo Clinic facilities in Minnesota, Florida, and Arizona. We reviewed all culture-positive microbiologic specimens of *Nocardia* spp. during December 2011–November 2018. A previous study from Mayo Clinic Florida published clinical outcomes for patients with invasive nocardiosis during 1998–2018 (*13*); the Florida cohort for this study included all patients identified during December 2011–November 2018 from that study. We defined invasive nocardiosis diagnosis as culture-positive for *Nocardia* spp. together with clinical or radiographic evidence of organ involvement. We defined disseminated disease if infection was identified by culture or radiographic imaging in >2 noncontiguous organs, with *Nocardia* isolated from >1 site, or by a single positive blood culture. 

### Demographic and Clinical Data

We extracted information from electronic medical records about demographics, coexisting conditions, and antimicrobial drugs used. We classified as transplant recipients those patients who had received a solid organ or hematopoietic stem cell transplant before *Nocardia* infection was diagnosed. We referred to antimicrobial drugs administered soon after *Nocardia* spp. was isolated or detected on special stains as initial therapy. After species confirmation and antimicrobial susceptibility testing results became available, we recorded subsequent treatment as definitive therapy. Outcome data included all-cause mortality at 1 year after diagnosis of invasive nocardiosis. 

Patients received an initial diagnosis of nocardiosis, an evaluation, and treatment at 1 of 3 sites: Mayo Clinic Rochester (Minnesota), Mayo Clinic Arizona in Phoenix/Scottsdale, Arizona (Arizona), or Mayo Clinic Florida in Jacksonville, Florida (Florida). We extracted postal (ZIP) code–specific location data for all participants based on residence at the time of data extraction. Clinical outcomes for the Florida cohort include data for the December 2011–November 2018 subset of patients from the earlier Mayo Clinic study (*13*). 

### Microbiology

We extracted information on type of specimen, species, and antimicrobial susceptibilities for *Nocardia* isolates from the microbiology database at Mayo Clinic Laboratories (https://www.mayocliniclabs.com). We categorized species as other if <5 isolates of that species were identified or if specific species was undetermined. We identified *Nocardia* at the species level using matrix-assisted laser desorption/ionization time-of-flight mass spectrometry (MALDI-TOF MS) analysis and if necessary, 16s ribosomal RNA sequencing. Antimicrobial susceptibility testing using broth microdilution was performed at Mayo Clinic Laboratories. 

Of note, 3 isolates from 2012–2015 in the study were identified as either *N. asteroides* (*2*) or *N. asteroides* complex (*1*). Because of taxonomic changes in *Nocardia* identification on the basis of molecular methodologies, *N. asteroides* complex has been reclassified into 6 taxa, with *N. cyriacigeorgica* (drug pattern VI) being the most common (*11*). Because we could not reclassify those 3 isolates, we included them in the other species category. 

### Statistical Analysis

We described patient demographics, coexisting conditions, disease characteristics, and outcomes for the overall cohort and for subgroups by geographic location of treatment (Minnesota, Arizona, or Florida) and by transplant status, using frequency and percentages for categorical variables and means and SDs or medians and interquartile ranges (IQRs) for continuous variables. We plotted patient residential ZIP codes and frequency of *Nocardia* spp. by geographic location of treatment on a map of the United States. We also described initial empiric antimicrobial therapy and subsequent treatment after identification of *Nocardia* spp. and antimicrobial susceptibilities were available. We performed all analyses using SAS version 9.4 (SAS Institute). 

## Results

Of 268 patients with invasive nocardiosis during the study period, 129 (48.2%) were enrolled at the Mayo Clinic site in Minnesota, 87 (32.4%) in Arizona, and 52 (19.4%) in Florida. We recorded data on demographics, coexisting conditions, and organs transplanted by study site (Table 1). *N. nova* complex, 43 (33.4%) cases, was the predominant species in Minnesota and *N cyriacigeorgica*, 36 (41.4%) cases, in Arizona. In Florida, the most common species were *N. brasiliensis*, 9 (17.3%) cases, and *N. cyriacigeorgica* and *N. farcinica*, 8 (15.4%) cases each (Table 2). Other species accounted for 12.4% of cases in Minnesota, 8% in Arizona, and 26.9% in Florida. In Arizona, *N. wallacei* accounted for 13.8% and *N. transvalensis* complex for 11.5% of isolates, but they each constituted <2% of isolates in Florida and Minnesota. 

**Table 1:** Patient demographics and coexisting conditions by site of diagnosis and transplant status in study of invasive *Nocardia* infections across 3 distinct geographic regions, United States*

Category		Site of diagnosis					Transplant recipient		Total, n = 268
		MN, n = 129	AZ, n = 87		FL, n = 52		No, n = 186	Yes, n = 82	
Demographics									
Age, mean (SD)		63.4 (16.3)	65.9 (15.0)		61.2 (11.7)		66.1 (15.7)	58.6 (12.4)	63.8 (15.1)
Sex									
M		61 (47.3)	41 (47.1)		37 (71.2)		83 (44.6)	56 (68.3)	139 (51.9)
F		68 (52.7)	46 (52.9)		15 (28.8)		103 (55.4)	26 (31.7)	129 (48.1)
White		121 (97.6)	71 (84.5)		39 (76.5)		173 (96.6)	58 (72.5)	231 (89.2)
Hispanic		0 (0.0)	12 (14.0)		3 (5.9)		4 (2.2)	11 (13.4)	15 (5.7)
Coexisting conditions									
Diabetes		7 (5.4)	18 (20.7)		23 (44.2)		12 (6.5)	36 (43.9)	48 (17.9)
Liver failure		4 (3.1)	1 (1.1)		2 (3.8)		1 (0.5)	6 (7.3)	7 (2.6)
Renal failure/dialysis		6 (4.7)	4 (4.6)		17 (32.7)		2 (1.1)	25 (30.5)	27 (10.1)
Active malignancy		16 (12.5)	12 (13.8)		13 (25.0)		26 (14.0)	15 (18.5)	41 (15.4)
Rheumatologic conditions		21 (16.4)	5 (5.7)		7 (13.5)		28 (15.1)	5 (6.2)	33 (12.4)
Chronic lung disease		95 (74.8)	34 (39.1)		27 (51.9)		130 (69.9)	26 (32.5)	156 (58.6)
Transplant recipient		14 (10.9)	35 (40.2)		33 (63.5)		NA	NA	82 (30.6)
Transplanted organs									
Kidney		2 (14.3)	18 (51.4)		13 (39.4)		NA	33 (40.2)	33 (40.2)
Liver		2 (14.3)	2 (5.7)		1 (3.0)		NA	5 (6.1)	5 (6.1)
Pancreas		1 (7.1)	0		0		NA	1 (1.2)	1 (1.2)
Lung		1 (7.1)	0†		12 (36.4)		NA	13 (15.9)	13 (15.9)
Heart		2 (14.3)	9 (25.7)		2 (6.1)		NA	13 (15.9)	13 (15.9)
Kidney and liver		1 (7.1)	0		1 (3.0)		NA	2 (2.4)	2 (2.4)
Kidney and pancreas		0	0		3 (9.1)		NA	3 (3.7)	3 (3.7)
HSCT		5 (35.7)	6 (17.1)		1 (3.0)		NA	12 (14.6)	12 (14.6)

*Values are no. (%) patients except at indicated. AZ, Mayo Clinic Arizona, Phoenix/Scottsdale, Arizona, USA; FL, Mayo Clinic Florida, Jacksonville, Florida, USA; HSCT, hematopoietic stem cell transplant; MN, Mayo Clinic Rochester, Rochester, Minnesota, USA; NA, not applicable. †Lung transplantations not performed at Mayo Clinic Arizona during study period.

**Table 2:** Disease characteristics and outcomes by site of diagnosis and transplant status in study of invasive *Nocardia* infections across 3 distinct geographic regions, United States*

Disease characteristics	Site of diagnosis		Transplant recipient	Total, n = 268

MN, n = 129	AZ, n = 87	FL, n = 52	No, n = 186	Yes, n = 82
Diseased organ							
Lung only	106 (82.2)	80 (92.0)	31 (59.6)		157 (84.4)	60 (73.2)	217 (81.0)
Skin/soft tissue only	8 (6.2)	0 (0.0)	10 (19.2)		11 (5.9)	7 (8.5)	18 (6.7)
Disseminated	15 (11.6)	7 (8.0)	11 (21.2)		18 (9.7)	15 (18.3)	33 (12.3)
Any CNS involvement	6 (4.7)	5 (5.7)	5 (9.6)		6 (3.2)	10 (12.2)	16 (6.0)
*Nocardia* spp.							
* N. abscessus*	13 (10.1)	1 (1.1)	3 (5.8)		14 (7.5)	3 (3.7)	17 (6.3)
* N. brevicatena/N. paucivorans*	3 (2.3)	2 (2.3)	0		2 (1.1)	3 (3.7)	5 (1.9)
* N. cyriacigeorgica*	24 (18.6)	36 (41.4)	8 (15.4)		52 (28.0)	16 (19.5)	68 (25.4)
* N. farcinica*	26 (20.2)	13 (14.9)	8 (15.4)		30 (16.1)	17 (20.7)	47 (17.5)
* N. nova complex*	43 (33.3)	2 (2.3)	6 (11.5)		44 (23.7)	7 (8.5)	51 (19.0)
* N. transvalensis complex*	1 (0.8)	10 (11.5)	1 (1.9)		10 (5.4)	2 (2.4)	12 (4.5)
* N. brasiliensis*	2 (1.6)	2 (2.3)	9 (17.3)		8 (4.3)	5 (6.1)	13 (4.9)
* N. wallacei*	0	12 (13.8)	1 (1.9)		7 (3.8)	6 (7.3)	13 (4.9)
* N. veterana*	1 (0.8)	2 (2.3)	2 (3.8)		0	5 (6.1)	5 (1.9)
Other†	16 (12.4)	7 (8.0)	14 (26.9)		19 (10.2)	18 (22.0)	37 (13.8)
Outcomes							
Alive after 1 y	108 (91.5)	69 (83.1)	44 (84.6)		155 (90.6)	66 (80.5)	221 (87.4)
Died within 1 y	21 (8.5)	18 (16.9)	8 (15.4)		31 (9.4)	16 (19.5)	47 (12.6)

*Values are no. (%) patients. AZ, Mayo Clinic Arizona, Phoenix/Scottsdale, Arizona, USA; CNS, central nervous system; FL, Mayo Clinic Florida, Jacksonville, Florida, USA; MN, Mayo Clinic Rochester, Rochester, Minnesota, USA. †Other species (n<5 each): *N. amikacinitolerans* (1), *N. asiatica* (1), *N. asteroides* (3), *N. beijingensis* (3), *N. carnea* (1), *N. cerradoensis* (1), *N. exalbida* (1), *N. flavorosea* (1), *N. kruczakiae* (1), *N. niwae* (2), *N. otitidiscaviarum* (1), *N. pseudobrasiliensis* (4), *N. puris* (1), *N. takedensis* (3), *N. vermiculata* (1), *N. vinacea* (1), *N. yamanashiensis* (1); no species identified (9).

Of the 268 patients, 82 (30.6%) were transplant recipients; 70 received solid organ and 12 hematopoietic stem cell transplants. Median time from transplant to diagnosis of invasive nocardiosis was 12 months (IQR 5–54 months). Kidneys, 33 (40.2%) cases, were the most common transplanted organ; more than half (17/33, 51%) of patients underwent transplantation in Arizona. Most heart recipients (9/13, 69%) also underwent transplantation in Arizona. Most (12/13, 92%) lung recipients underwent transplantation in Florida; however, during the study period, lung transplants were not performed at Mayo Clinic Arizona. Overall mean age of all transplant recipients was 63.8 years (SD 15.1). Chronic lung disease (58.6%) and diabetes (17.9%) were 2 of the most common coexisting conditions (Table 1). 

We found isolated nocardial pulmonary involvement in 84.4% of non–transplant patients and 73.2% of transplant patients. Rate of dissemination was 12.3% in the total cohort: 9.7% in non–transplant patients, 18.3% in transplant patients (Table 2). We documented central nervous system (CNS) involvement in 12.2% of transplant recipients and 3.2% of non–transplant patients. (Table 2). The most commonly identified species among transplant recipients were *N. farcinica* (20.7%, n = 17) and *N. cyriacigeorgica* (19.5%, n = 16) and among non–transplant patients, *N. cyriacigeorgica* (38.0%, n = 52), *N. nova* complex (23.7%, n = 44), and *N. farcinica* (16.1%, n = 30). 

When we analyzed the involvement of diseased organs in transplant patients, we found that *Nocardia* caused isolated pulmonary disease in 60/82 (75.0%) case-patients. Isolated lung disease was the predominant manifestation for most species: *N. cyriacigeorgica* caused isolated lung disease in 86.7% of transplant patients and combined lung and CNS disease in 13.3% of cases, and *N. farcinica* resulted in isolated lung disease in 76.5% of transplant patients and disseminated disease to the lungs, CNS, and skin/soft tissue in 11.8% of patients. Similarly, *N. nova* complex caused isolated pulmonary disease in 83.3% and isolated skin/soft tissue disease in 16.7%. In contrast, *N. brasiliensis* resulted in isolated skin/soft tissue disease in 60% of transplant patients and isolated lung disease in only 20%. Only 16/268 (6.0%) of all patients and 10/82 (12.2%) transplant patients had nocardial CNS involvement; *N. farcinica* (42.8%, n = 6), *N. cyriacigeorgica* (14.3%, n = 2), and *N. wallacei* (14.3%, n = 2) were the species most often associated with CNS disease. Rates of CNS involvement among transplant patients were similar across all 3 sites: 4.7% in Minnesota, 5.7% in Arizona, and 9.6% in Florida. 

Antimicrobial susceptibilities varied among the most commonly isolated *Nocardia* spp. (Table 3). All *Nocardia* isolates were 100% susceptible to linezolid. Analysis of species-specific patterns of susceptibility revealed almost no differences by geographic location in susceptibility to antimicrobials across the 3 Mayo Clinic sites. Trimethoprim/sulfamethoxazole (TMP/SMX) susceptibility was 99.5% among non–transplant patients, 96.3% among transplant recipients, and 99% for the total cohort. Exceptions among *Nocardia* spp. susceptibility to TMP/SMX were 94% for *N. abscessus* and 92.0% for those listed collectively as other species. Susceptibility to ceftriaxone ranged from 0% for *N. brasiliensis* to 100% for *N. brevicatena*/*N. paucivorans*. Similarly, susceptibility to imipenem varied; *N. cyriacigeorgica* (97%), *N. nova* complex (98%), and *N. brevicatena/N. paucivorans* (100%) were the most susceptible species (Table 3). For all antimicrobial drugs administered during initial empiric therapy, we calculated the percentage retained in subsequent treatment regimens after analysis of *Nocardia* antimicrobial susceptibilities (Table 4). 

**Table 3:** Antimicrobial susceptibilities based on *Nocardia* spp. in study of invasive *Nocardia* infections across 3 distinct geographic regions, United States*

Species	No. isolates	Susceptibility												
		AM/CL	CEF	CTX	IMP	CIP	MOX	CLA	AMI	TOB	DOX	MIN	TMP/SMX	LIN
*N. abscessus*	17	82	82	94	59	0	14	41	100	94	88	88	94	100
*N. brevicatena/N. paucivorans*	5	50	75	100	100	100	100	100	100	100	100	100	100	100
*N. cyriacigeorgica*	68	6	28	60	97	2	3	2	99	99	6	9	100	100
*N. farcinica*	47	96	4	4	87	57	88	4	100	0	0	6	100	100
*N. nova complex*	51	4	51	12	98	4	7	100	98	6	2	18	100	100
*N. transvalensis complex*	12	92	17	58	8	58	100	0	8	0	0	17	100	100
*N. brasiliensis*	13	100	0	0	15	0	46	0	100	100	8	0	100	100
*N. wallacei*	13	92	8	67	0	58	100	0	25	0	17	50	100	100
*N. veterana*	5	0	60	20	100	0	0	100	100	20	0	20	100	100
Other†	37	11	44	61	78	42	61	50	94	86	47	67	92	97
Total	268	40	33	40	78	24	40	33	91	51	17	27	99	100

*AM/CL, amoxicillin/clavulanic acid; CEF, cefepime; CTX, ceftriaxone; IMP, imipenem; CIP, ciprofloxacin; MOX, moxifloxacin; CLA, clarithromycin; AMI, amikacin; TOB, tobramycin; DOX, doxycycline; MIN, minocycline; TMP/SMX, trimethoprim/sulfamethoxazole; LIN, linezolid †Other species (n<5 each): *N. amikacinitolerans* (1), *N. asiatica* (1), *N. asteroides* complex (3), *N. beijingensis* (3), *N. carnea* (1), *N. cerradoensis* (1), *N. exalbida* (1), *N. flavorosea* (1), *N. krucza*kiae (1), *N. niwae* (2), *N. otitidiscaviarum* (1), *N. pseudobrasiliensis* (4), *N. puris* (1), *N. takedensis* (3), *N. vermiculata* (1), *N. vinacea* (1), and *N. yamanashiensis* (1); no species identified (9)

**Table 4:** Choice of initial/empiric antimicrobials and subsequent antimicrobials after susceptibilities were reported in study of invasive *Nocardia* infections across 3 distinct geographic regions, United States*

Initial treatment	No. (%)	Treatment after species confirmation and antimicrobial susceptibility testing results														
		AM/ CL	CEF	CTX	IMP	CIP	MOX	CLA	AMI	TOB	DOX	MIN	TMP/ SMX	LIN	SUL	Other†
AM/CL	7 (2.6)	57	0	0	14	14	14	14	0	0	0	0	57	0	0	0
CEF	3 (1.1)	0	100	0	0	0	0	0	0	33	33	0	67	0	0	0
CTX	22 (8.2)	14	0	27	5	5	18	9	5	0	9	14	45	5	9	5
IMP	49 (18.3)	16	0	8	22	0	22	8	2	0	4	31	69	0	4	2
CIP	7 (2.6)	14	0	0	0	86	0	0	0	0	0	14	43	14	0	29
MOX	19 (7.1)	11	0	11	16	0	74	11	0	0	5	16	37	0	0	0
CLA	10 (3.7)	0	0	0	0	10	20	80	10	0	0	10	20	0	0	10
AMI	8 (3.0)	25	0	0	0	13	13	25	13	0	0	13	75	0	13	0
TOB	2 (0.8)	0	50	0	0	0	0	0	0	50	0	0	50	0	0	0
DOX	15 (5.6)	20	7	0	20	7	13	0	0	0	33	0	73	0	7	0
MIN	27 (10.1)	11	0	7	7	4	30	7	0	0	4	37	52	0	0	0
TMP/SMX	153 (57.1)	6	1	7	5	5	5	17	3	1	4	10	82	2	3	3
LIN	21 (7.8)	19	0	10	19	5	33	5	0	0	5	38	57	5	10	0
SUL	4 (1.5)	25	0	50		0	25	0	0	0	0	25	0	0	75	0
Other	15 (5.6)	7	0	7	7	13	0	0	0	0	13	13	60	0	0	13

*Gray shaded cells show percentages of patients in which initial antimicrobial was retained as part of subsequent therapy. AM/CL, amoxicillin/clavulanic acid; CEF, cefepime; CTX, ceftriaxone; IMP, imipenem; CIP, ciprofloxacin; MOX, moxifloxacin; CLA, clarithromycin; AMI, amikacin; TOB, tobramycin; DOX, doxycycline; MIN, minocycline; TMP/SMX, trimethoprim/sulfamethoxazole; LIN, linezolid, SUL, sulfadiazine †Other species (n<5 each): *N. amikacinitolerans* (1), *N. asiatica* (1), *N. asteroides* complex (3), *N. beijingensis* (3), *N. carnea* (1), *N. cerradoensis* (1), *N. exalbida* (1), *N. flavorosea* (1), *N. krucza*kiae (1), *N. niwae* (2), *N. otitidiscaviarum* (1), *N. pseudobrasiliensis* (4), *N. puris* (1), *N. takedensis* (3), *N. vermiculata* (1), *N. vinacea* (1), and *N. yamanashiensis* (1); no species identified (9).

We also analyzed overall *Nocardia* susceptibility patterns in transplant and non–transplant patients. Susceptibility was 51.9% for moxifloxacin and 32.1% for ciprofloxacin in transplant patients and 34.1% for moxifloxacin and 20.2% for ciprofloxacin in non–transplant patients. Imipenem was 72.8% susceptible in transplant patients and 80.3% in non–transplant patients; linezolid was 98.8% susceptible in transplant patients and 100.0% in non–transplant patients. TMP/SMX was 96.3% susceptible in transplant patients and 99.5% in non–transplant patients; ceftriaxone was 32.1% susceptible in transplant patients and 43.7% in non–transplant patients. Among nocardiosis patients, 155/186 (90.6%) non–transplant patients and 66/82 (80.5%) transplant recipients survived after 1 year. Rates of all-cause mortality at 1 year after diagnosis were similar for all 3 Mayo Clinic sites and different *Nocardia* spp. 

## Discussion

We identified 268 patients (82 transplant recipients and 186 non–transplant recipients) with invasive nocardiosis during 2011–2018 for this retrospective cohort study. Participants were patients at 3 Mayo Clinic tertiary care and high-volume transplant sites in Minnesota, Arizona, and Florida within the United States, which provided a unique opportunity to assemble a large cohort of *Nocardia* infected case-patients from within the entire Mayo Clinic database. 

We described clinical, microbiologic, drug susceptibility, and outcome data in patients with nocardiosis at each geographic site. *Nocardia* spp. varied by geographic site where diagnosis occurred, correlating with published differences in geographic distribution (*2*). The most common species of *Nocardia* isolated in Minnesota were *N. nova* complex, *N. farcinica*, and *N. cyriacigeorgica*. In Arizona, *N. cyriacigeorgica*, *N. farcinica*, and *N. wallacei* were the most common species. *N. brasiliensis* was the most common species in Florida, which might reflect the organism’s preference for tropical climates (*4*). Some patient demographics and clinical characteristics differed by site and might contribute to differences among species in occurrence, susceptibilities, and clinical outcomes.

Isolated lung involvement was the most common manifestation of *Nocardia* infection at all 3 sites: 92% in Arizona, 82% in Minnesota, and 60% in Florida. Possibly the arid climate of the desert southwest in Arizona presents greater opportunity for airborne dispersal of *Nocardia* and subsequent inhalation and pulmonary infection (similar to the transmission kinetics for coccidioidomycosis). Primary skin/soft tissue infection was present in 19.2% of cases in Florida, more common than in the other locations, likely related to a higher rate of *N. brasiliensis* infection (17.3%) in Florida; *N. brasiliensis* is known to cause isolated cutaneous infection (*3*,*14*). Patients in Florida had the highest rate of disseminated nocardiosis (21.2%). Rates of death at 1 year were similar among the 3 Mayo Clinic sites. Rates of death based on the species of *Nocardia* were also similar, which is contrary to a recent study indicating a higher death rate for *N. farcinica* (*15*). *N. farcinica* was most commonly associated with CNS involvement in our cohort, which is consistent with previous reports (*16*,*17*).

All *Nocardia* isolates in our study were susceptible to linezolid, in agreement with other studies (*18*,*19*). The 3 most common *Nocardia* spp. implicated in invasive infection in this study, *N. cyriacigeorgica*, *N. farcinica*, and *N. nova* complex, were 100% susceptible to TMP/SMX. Susceptibility profiles for >1,200 *Nocardia* isolates reported in another study also noted universal susceptibility to linezolid. TMP/SMX resistance was rare (2%), except among *N. pseudobrasilensis* (31%) and *N. transvalensis* complex (19%) (*20*). In our study, we noted only 3.7% resistance to TMP/SMX among *Nocardia* isolates in transplant recipients, which is in agreement with other studies that have reported low overall resistance rates (*21*–*23*). However, ongoing surveillance for such trends is paramount. In patients with *Nocardia* infection, especially those receiving substantial immune-suppression treatments, using combination antimicrobial therapy before availability of susceptibility testing results is imperative. Combination therapy should always include either linezolid or TMP/SMX. Based on our susceptibility data, an appropriate second antimicrobial choice would be imipenem, which has the added advantage of excellent CNS penetration. We also calculated the percentage of each antimicrobial in our initial empiric regimen that was retained in subsequent regimens after susceptibilities to all antimicrobials were known (Table 4). 

TMP/SMX dosage for *Pneumocystis jirovecii* pneumonia prophylaxis after organ transplantation is not necessarily protective against invasive *Nocardia* infection (*9*,*13*). Guidelines addressing *Nocardia* infections in solid organ transplantation suggest that TMP/SMX prophylaxis might be helpful in preventing primary *Nocardia* infection or relapse after treatment, although infections can occur despite prophylaxis. For secondary prophylaxis, a TMP/SMX dosage of 1 double-strength tablet daily (dosage-adjusted for renal function) has been offered as a consideration, although that recommendation was weak and supported by low-quality evidence (*16*).

A recent study reported a death rate after 1 year of 16.8% in solid organ transplant recipients with *Nocardia* infection. Independent risk factors for death included liver transplantation and time from symptom onset to seeking treatment; disseminated infection was not associated with increased death (*15*). Our study found death rates after 1 year of 19.5% among transplant recipients and 9.4% among non–transplant patients, an observation concordant with findings in a larger study from Europe of nocardiosis in solid organ transplant recipients (*24*). However, another recent publication noted similar death rates for transplant and non–transplant recipients (*13*). Retrospective designs, different underlying risk factors for infection, and variable definitions used in published studies make drawing firm conclusions difficult. 

Among the limitations of our study, we did not collect detailed clinical or radiographic results or match specific treatment information with outcomes. However, those data have been elaborated in previous studies, including from Mayo Clinic (*13*,*15*). The primary purposes of our study were to determine regional differences among patients with risk factors, geographic distribution of *Nocardia* spp., organs involved by geographic location and species of *Nocardia*, and differences in antimicrobial susceptibility. Second, even though patients were from distinct geographic locations, they were all primarily managed within the Mayo Clinic system. Patients from other regions and institutions were not represented. However, the substantial climatic and environmental differences among the 3 regions studied—midwestern, southeastern, and southwestern United States—led us to decide on identifying unique trends in *Nocardia* spp. and disease manifestations for those study sites. A larger prospective study encompassing patients and institutions from across the United States and other parts of the world could uncover additional nuances in data pertaining to both pathogens and hosts. Third, data on residential ZIP codes of patients (Figure) was collected at time of data extraction and may differ slightly from the residences of patients at time of diagnosis and treatment. Mayo Clinic’s research data platform does not store historical residential data. However, the primary geographic variable used for analysis was site of diagnosis and treatment; ZIP codes were used only to illustrate the large regional representation of patients seeking treatment at Mayo Clinic sites as destination healthcare centers. It is worth noting that 88.2% of patients still resided within the region of diagnosis and treatment at time of data extraction, which further validated our conclusions about geographic distribution and variability of *Nocardia* spp*.* Fourth, univariate analysis of 1-year survival did not account for possible confounders in the relationship between transplant status and all-cause death. Fifth, we did not record the number of *Nocardia* isolates excluded because of lack of clinical or radiographic evidence representing potential *Nocardia* colonization. Sixth, we collected limited data on coexisting conditions and socioeconomic factors and did not record specifics of immune suppression besides organ transplantation. Finally, we recognize inherent biases associated with retrospective studies, such as selection bias and center effect.

**Figure:**
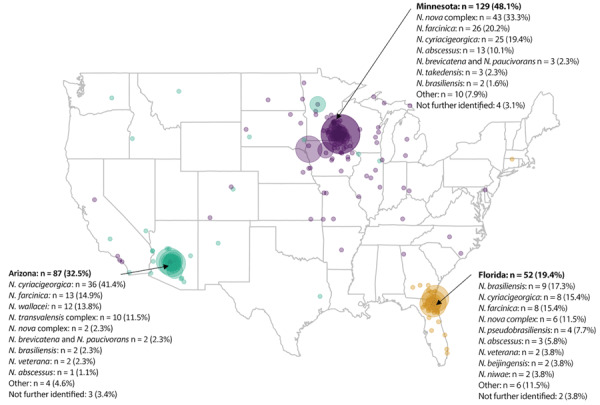
Geographic distribution of patients for study of invasive *Nocardia* infections across 3 distinct geographic regions, United States. Color codes and list of *Nocardia* spp. represent the geographic location of diagnosis and treatment. Each circle represents a patient in the study cohort’s postal (ZIP) code of residence at the time of data extraction (2022); larger circles represent ZIP codes with more patients. Percentages for states are for the full study cohort; percentages for individual species are for that state.

In summary, our study provides information on differences in geographic distribution, patient characteristics, disease manifestations, and antimicrobial susceptibility patterns related to *Nocardia* spp. in noncontiguous regions of the United States with varied climatic conditions. Similar investigations of patients with invasive infections caused by pathogens of environmental origin encompassing broader geographic regions from around the world would help continue to expand knowledge about how to manage and treat such infections effectively. 
